# Luteolin ameliorates loperamide-induced functional constipation in mice

**DOI:** 10.1590/1414-431X2023e12466

**Published:** 2023-01-27

**Authors:** Yujin Wang, Hua Jiang, Lijun Wang, Huiping Gan, Xinchun Xiao, Liangwu Huang, Wenxin Li, Zongrun Li

**Affiliations:** 1 College of Basic Medicine, Shaanxi University of Chinese Medicine, Xianyang, Shaanxi, China; 2 The First Clinical Medical College, Shaanxi University of Chinese Medicine, Xianyang, Shaanxi, China

**Keywords:** Functional constipation, Luteolin, Intestinal motility, Interstitial cells of Cajal, AQPs

## Abstract

Functional constipation (FC) is one of the most common gastrointestinal disorders characterized by hard stools and infrequent bowel movements, which is associated with dysfunction of the enteric nervous system and intestinal motility. Luteolin, a naturally occurring flavone, was reported to possess potential pharmacological activities on intestinal inflammation and nerve injury. This study aimed to explore the role of luteolin and its functional mechanism in loperamide-induced FC mice. Our results showed that luteolin treatment reversed the reduction in defecation frequency, fecal water content, and intestinal transit ratio, and the elevation in transit time of FC models. Consistently, luteolin increased the thickness of the muscular layer and lessened colonic histopathological injury induced by loperamide. Furthermore, we revealed that luteolin treatment increased the expression of neuronal protein HuC/D and the levels of intestinal motility-related biomarkers, including substance P (SP), vasoactive intestinal polypeptide (VIP), and acetylcholine (ACh), as well as interstitial cells of Cajal (ICC) biomarker KIT proto-oncogene, receptor tyrosine kinase (C-Kit), and anoctamin-1 (ANO1), implying that luteolin mediated enhancement of colonic function and contributed to the anti-intestinal dysmotility against loperamide-induced FC. Additionally, luteolin decreased the upregulation of aquaporin (AQP)-3, AQP-4, and AQP-8 in the colon of FC mice. In summary, our data showed that luteolin might be an attractive option for developing FC-relieving medications.

## Introduction

Functional constipation (FC), defined as constipation without a known physiological or anatomical cause, is a common clinical condition that negatively affects the quality of life of 10-15% of the population in the world (1). It is worth noting that the prevalence of FC in China has increased from 5.5 to 10.9% over the past three decades (2). FC is a functional disorder characterized by persistent, infrequent, difficult, or incomplete defecation and a sensation of anorectal obstruction (3). In addition to the physical health concerns associated with FC, particularly frequent pain, FC also affects the mental health of patients (4).

In general, dysfunctional gastrointestinal motility, as evidenced by prolonged colon transit time and fewer bowel movements, is the main pathogenic mechanism characteristic of FC (5). Multiple intestinal motility-related biomarkers, including substance P (SP), vasoactive intestinal polypeptide (VIP), acetylcholine (ACh), nitric oxide (NO), and others, are located in the myenteric and submucosal ganglia of the colon and provide a physiological balance in mediating the functions of the colon (6). As an excitatory neurotransmitter, SP is secreted by excitatory motor neurons and results in intestinal smooth muscle contraction. A decreased expression of VIP is associated with impaired rectal sensory function and colonic motility (7). ACh released from parasympathetic nerves mediates muscle contraction (8). Moreover, interstitial cells of Cajal (ICC) are involved in regulating autonomic nerves that predominantly facilitate intestinal motility (9). Notably, aquaporins (AQPs) are involved in the regulation of water metabolism in the intestinal tract (10). Because of the increasing incidence and complex pathogenic mechanism of FC, there is an urgent need to explore appropriate drugs for FC therapy.

Traditionally, FC patients are told to alter their diet and lifestyle in order to relieve the symptoms, but this approach has limited effect. In current care, many patients with FC receive symptomatic therapies with laxatives, which do not always achieve adequate therapeutic efficacy (11). Therefore, it is crucial to explore more efficacious drugs that can recover the intestinal function and improve gut motility and several metabolic parameters.

Luteolin is the primary member of the flavone family present in several vegetables and fruits. Previous studies reported that luteolin had extensive pharmacological and biological properties, including neuroprotective and antioxidant activities (12). Luteolin protected against intestinal inflammation both in cellular models and in mice (13,14). Luteolin had particularly powerful effects in alleviating diarrhea by mitigating the pathological condition of small intestine tissue and maintaining electrolyte balance (15,16). Furthermore, luteolin was reported to suppress NO production (17). Nevertheless, the effects of luteolin on intestinal motility in FC are not yet well understood. We hypothesized that luteolin is a potential drug and has an important role in FC therapy.

To investigate the potential mechanism associated with the recovery of the colon function in FC, we explored whether the constipating effects of loperamide administered to ICR mice may be alleviated by luteolin, which improves intestinal motility. Furthermore, because FC has multiple factors, ICC biomarkers and the levels of AQPs were determined in the colon of FC mice. In the present study, we aimed to reveal the possibility of luteolin alleviating FC.

## Material and Methods

### Animals

Male ICR mice purchased from Beijing HFK Bioscience Co., Ltd. (China) were grouped into four groups: control, FC, lactulose, and luteolin. The mice from the luteolin group and the lactulose group were treated orally with luteolin (80 mg/kg in 0.5% carboxymethyl cellulose sodium, Aladdin, China) or lactulose (500 mg/kg, Aladdin), respectively, daily for 14 days. To induce FC, loperamide (5 mg/kg, Aladdin) was administered orally to the above mice from day 12 to 14 (18). The mice from the control group received an equal volume of pure water. After treatment, defecation count, fecal mass, and body weight were recorded. The feeding behavior, such as water and food intake, was measured using a metabolic cage. The experimental procedures followed the Animal Care and Use Committee of Shaanxi University of Chinese Medicine (SUCMDL20190314001).

### Intestinal transit in mice

After 14 days of treatment, overnight fasted mice were orally given 0.1 mL of 20% charcoal (Aladdin) and 10% gum arable (Aladdin). Mice were sacrificed 30 minutes later, and the intestines were removed from the rectum through the pylorus. The charcoal meal traveled in the intestine was used to calculate intestinal transit ratio as follows: Intestinal transit ratio (%) = length traveled by the charcoal meal / total length of intestine × 100. A solution of Evans blue (5%, Sigma-Aldrich, USA) suspended in 1.5% methyl cellulose (Aladdin) was administered by gavage to mice. Feces were monitored at 10-min intervals for the presence of Evans blue. The transit time was recorded as the interval between the time at which gavage took place and the time that Evans blue was firstly observed in fecal pellets. These methodologies were performed according to previous studies (19,20).

### Histopathological analysis

Fixed distal colon tissues were embedded in paraffin, cut into 5-μm consecutive sections, and stained with hematoxylin & eosin (HE) using standard procedures. The histopathological features of the stained sections were observed using an Olympus DP73 microscope (Olympus, Japan).

The expression and distribution of KIT proto-oncogene, receptor tyrosine kinase (C-Kit), and anoctamin-1 (ANO1) was assessed using immunofluorescence staining. Briefly, after embedding in paraffin, fixed distal colon tissues were cut into 5-μm sections. Each section was rehydrated, blocked with 1% BSA at room temperature for 15 min, and incubated with primary antibodies for ANO1, C-Kit (1:100, Affinity Biosciences, China), or HuC/D (1:200, ThermoFisher Scientific, USA) at 4°C overnight. Subsequently, the sections were washed with PBS and incubated with secondary antibodies conjugated to Cy3 (1:200, Invitrogen, USA) at room temperature for 60 min followed by evaluation of the protein expressions using a fluorescence microscope (Olympus).

Immunohistochemical analysis was performed to test the histological distribution of AQP4. After antigen retrieval, incubation with 3% hydrogen peroxide and blocking with 1% BSA, sections were incubated with anti-AQP4 antibody (1:50, Affinity Biosciences) and goat anti-rabbit horse-radish peroxidase (HRP)-conjugated antibody (1:500, ThermoFisher Scientific). A 3,3′-diaminobenzidine (DAB, MXB Biotechnologies, China) substrate was used to visualize the antigen-antibody complexes.

### Measurement of gastrointestinal motility-related biomarkers

The concentration of SP and VIP were quantified using ELISA kits (FineTest, China) according to the manufacturer's instructions. The levels of NO and ACh in the colon tissues were determined using commercial ELISA kits (Jiancheng Bioengineering Institute, China).

### Western blotting analysis

Total proteins extracted from the colon tissues were quantified using a BCA protein assay kit (Beyotime Biotech Co., Ltd., China), separated by SDS-PAGE, and transferred to PVDF membranes (ThermoFisher Scientific). The membranes were incubated with the following primary antibodies: inducible nitric oxide synthase (iNOS), AQP-3 (1:1000, ABclonal, China), C-Kit, ANO1, AQP-4, and AQP-8 (1:1000, Affinity Biosciences) at 4°C overnight. After washing using TBST, membranes were reacted with HRP-conjugated antibodies (1:10000, ProteinTech, USA) at 37°C for 40 min. The bands were monitored with the ECL-Reagent (7 Sea Biotech, China) and visualized with a gel imaging analysis system (LIUYI, China).

### Statistical analysis

Numerical data are reported as means±SD. The significance of observed differences in the means was evaluated by ANOVA and Tukey's multiple comparisons test. Results with P<0.05 were considered statistically significant.

## Results

### Luteolin alleviated loperamide-induced FC in mice

**Figure 1:**
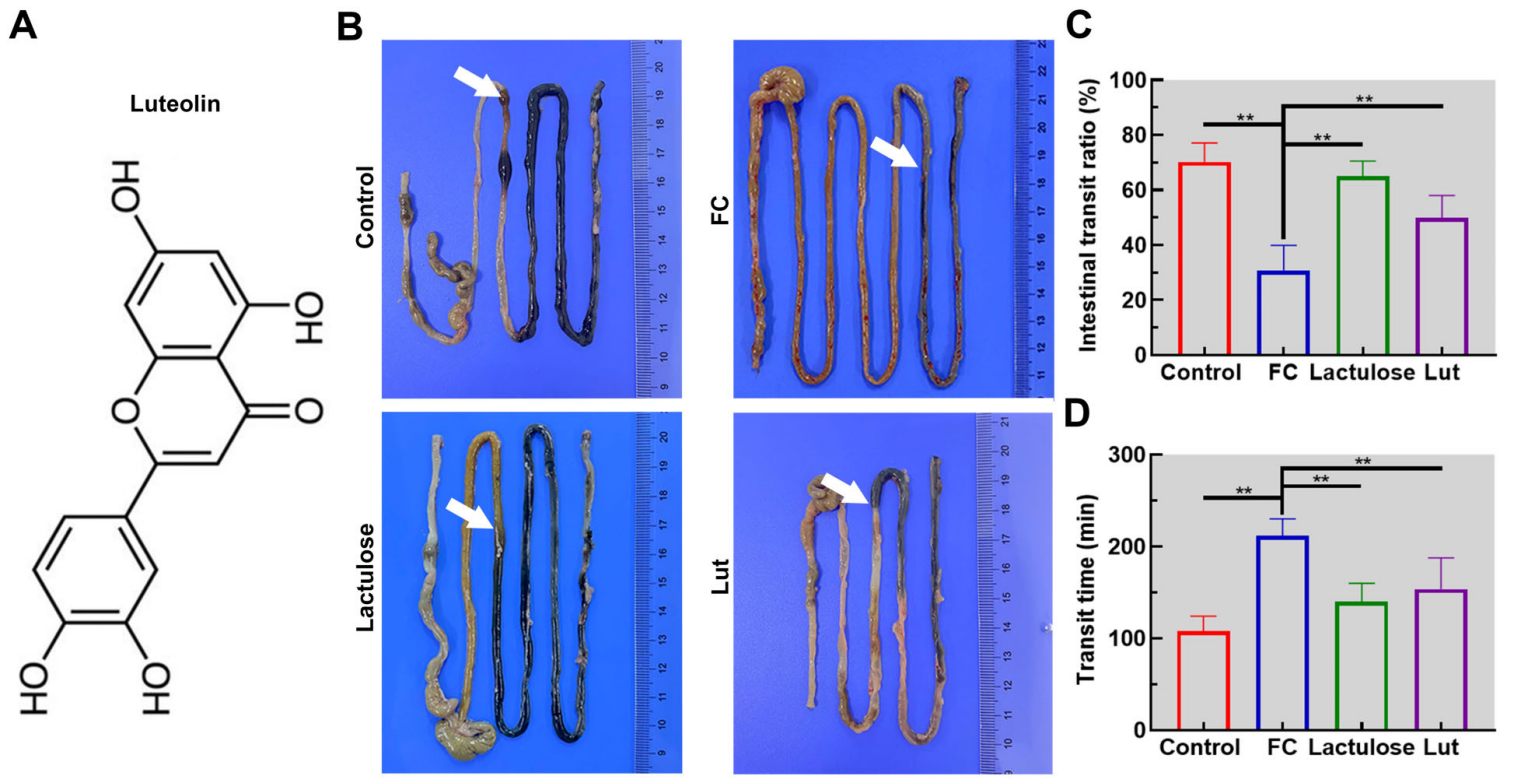
Luteolin (Lut) accelerated the intestinal transit in mice with functional constipation (FC). **A**, The structural formula of luteolin. **B**, Photographs of the intestinal tract (reference in cm). Intestinal transit ratio (**C**) and transit time (**D**) of mice. Data are reported as means±SD (n=6). **P<0.01 (ANOVA and Tukey's multiple comparisons test).

To induce FC, mice were intragastrically given loperamide for 3 days. Changes in body weight, feeding behavior, and feces of mice are shown in Table 1. Loperamide significantly decreased defecation count and fecal water content in mice (P<0.05). Nevertheless, almost no variation in body weight and feeding behavior were observed. In addition, loperamide notably decreased the intestinal transit ratio from 70.20 to 30.83% and delayed the transit time of mice from 107.67 to 211.67 min (Figure 1, P<0.01). However, these changes were found to be efficiently reversed in mice treated with luteolin. These findings showed that luteolin treatment mitigated FC in mice.

**Table 1:** Body weight, feeding behavior, and number of feces in mice with functional constipation (FC).

	Control	FC	Lactulose	Luteolin
Body weight (g)				
Day 12	35.33±1.37	33.67±0.82	33.17±1.94	33.67±1.75
Day 13	35.67±1.03	34.33±0.82	33.50±1.22	34.00±1.41
Day 14	35.83±1.17	34.50±0.84	34.17±1.60	34.33±1.37
Day 15	36.33±0.82	34.67±1.37	34.50±1.52	34.50±1.64
Feeding behavior				
Water intake (mL/24 h)	6.17±0.98	6.67±1.03	7.00±0.63	7.00±0.89
Food intake (g/24 h)	5.50±1.38	5.33±0.82	5.50±0.84	5.33±0.52
Feces				
Number	19.33±3.83	2.83±0.75**	11.33±2.25^^	6.17±1.17
Weight (g)	0.52±0.11	0.13±0.03**	0.29±0.06^^	0.26±0.07^
Water content (%)	68.64±5.74	50.41±8.49*	62.23±12.06	61.87±11.03

Data are reported as means±SD. *P<0.05, **P<0.01 compared with the control group; ^P<0.05, ^^P<0.01 compared with the FC group (ANOVA and Tukey's multiple comparisons test).

### Luteolin mitigated the histopathological injury in the colon

**Figure 2:**
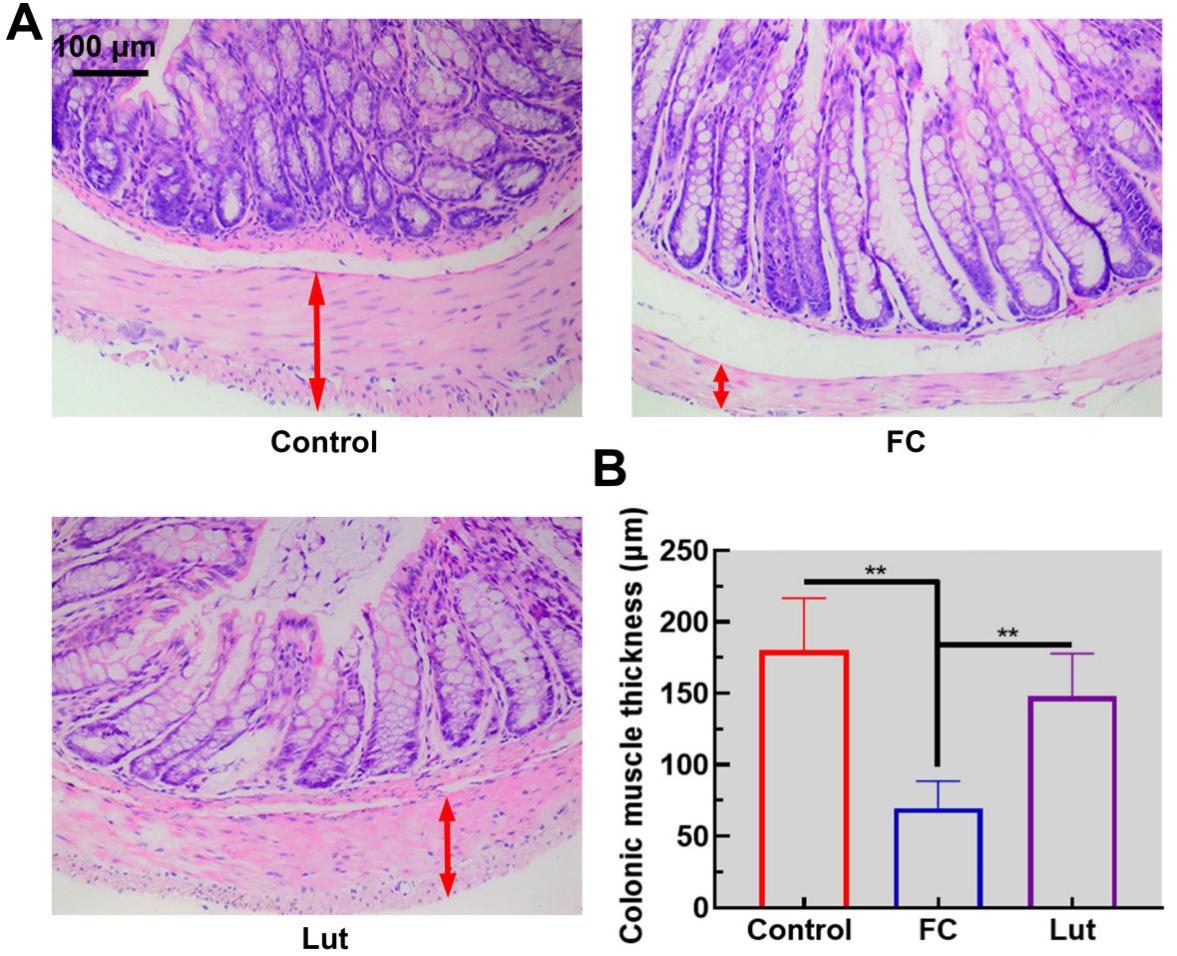
Luteolin (Lut) attenuated the pathological injury in the colon of mice with functional constipation (FC). **A**, Histopathological examination of the colon tissue following hematoxylin & eosin staining (scale bar 100 μm). **B**, Quantification of the colonic muscle thickness (double red arrows point out the muscular layer). Data are reported as means±SD (n=6). **P<0.01 (ANOVA and Tukey's multiple comparisons test).

Histopathological examinations of the colon showed that the thickness of the muscular layer was prominently decreased in FC mice, compared with the control, to approximately one-third of the baseline level (Figure 2, double red arrows indicate the muscular layer, P<0.01). Furthermore, a significant decrease in the number of goblet cells and enterocytes in the FC group was observed compared with the control group. Luteolin administration significantly improved the histopathological structure. Here, we demonstrated that luteolin alleviated colon injury in mice with FC.

### Luteolin regulated the neuronal protein and intestinal motility-related biomarkers

**Figure 3:**
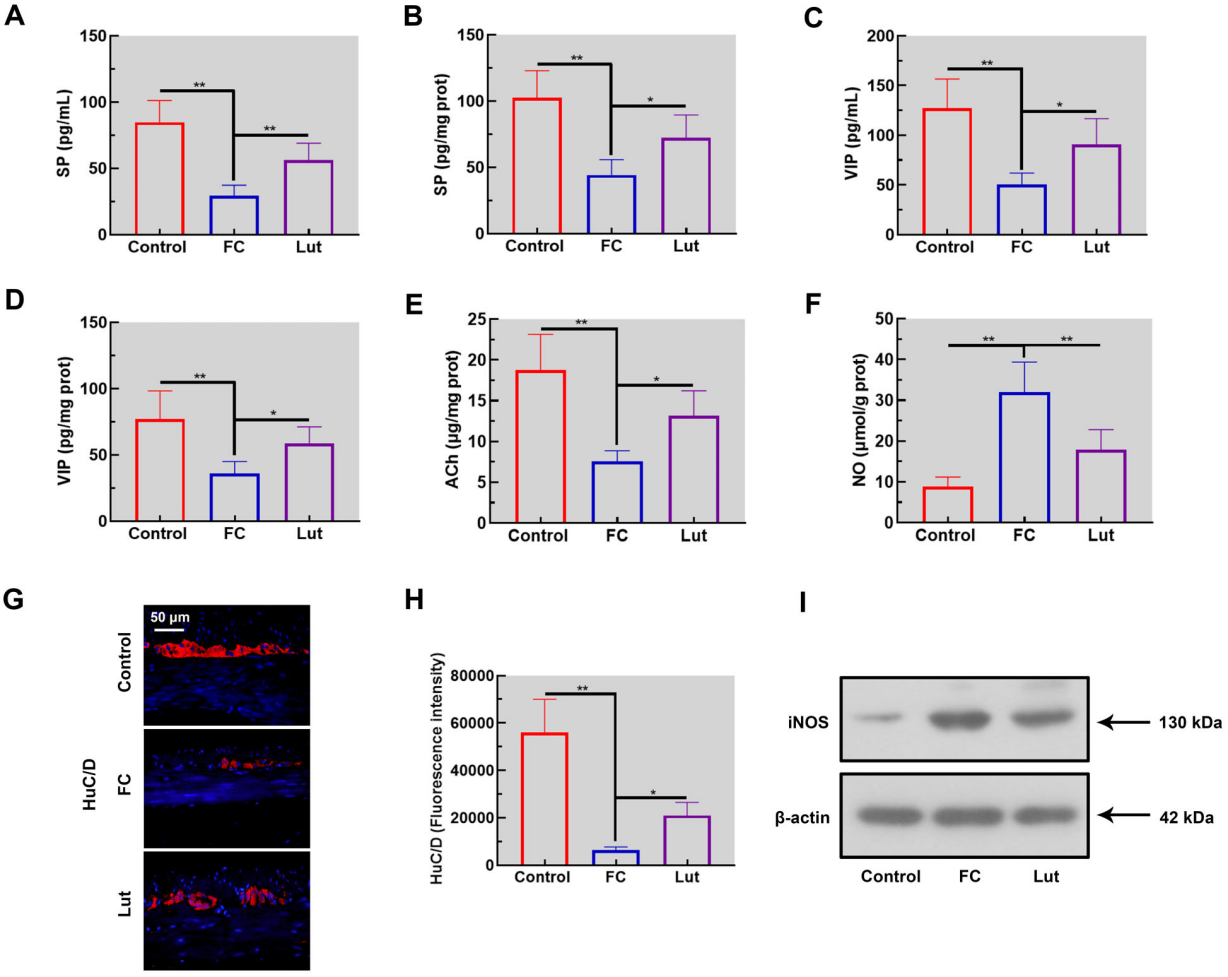
Luteolin (Lut) altered the levels of the neuronal protein and intestinal motility-related biomarkers of mice with functional constipation (FC). Levels of substance P (SP) in serum (**A**) and colon tissue (**B**). Concentrations of vasoactive intestinal polypeptide (VIP) in serum (**C**) and colon tissue (**D**). Levels of acetylcholine (ACh) (**E**) and nitric oxide (NO) (**F**) in the colon. The expression of HuC/D was evaluated by immunofluorescence assay (**G** and **H**). Scale bar 50 μm. **I**, Western blotting analysis of inducible nitric oxide synthase (iNOS) in the colon tissue. Data are reported as means±SD (n=6). *P<0.05 and **P<0.01 (ANOVA and Tukey's multiple comparisons test).

Biomarkers related to intestinal motility were analyzed in serum and colon tissues of FC mice. Compared with the control mice, levels of SP, VIP, and ACh were decreased significantly in the serum and colon of FC mice (Figure 3A-E, P<0.01). HuC/D immunofluorescence-stained sections were used to detect neurons in the colon. As shown in Figure 3G and H, myenteric neurons displayed a scant HuC/D expression in FC mice (P<0.01). Conversely, NO concentration and iNOS expression were increased in the colon of FC mice induced by loperamide (Figure 3F and I). Remarkably, the above alterations were restored after luteolin administration. These results indicated that luteolin treatment regulated intestinal motility-related biomarkers as well as neurotransmission in the colon tissues of FC mice.

### Luteolin increased the levels of ICC biomarkers

**Figure 4:**
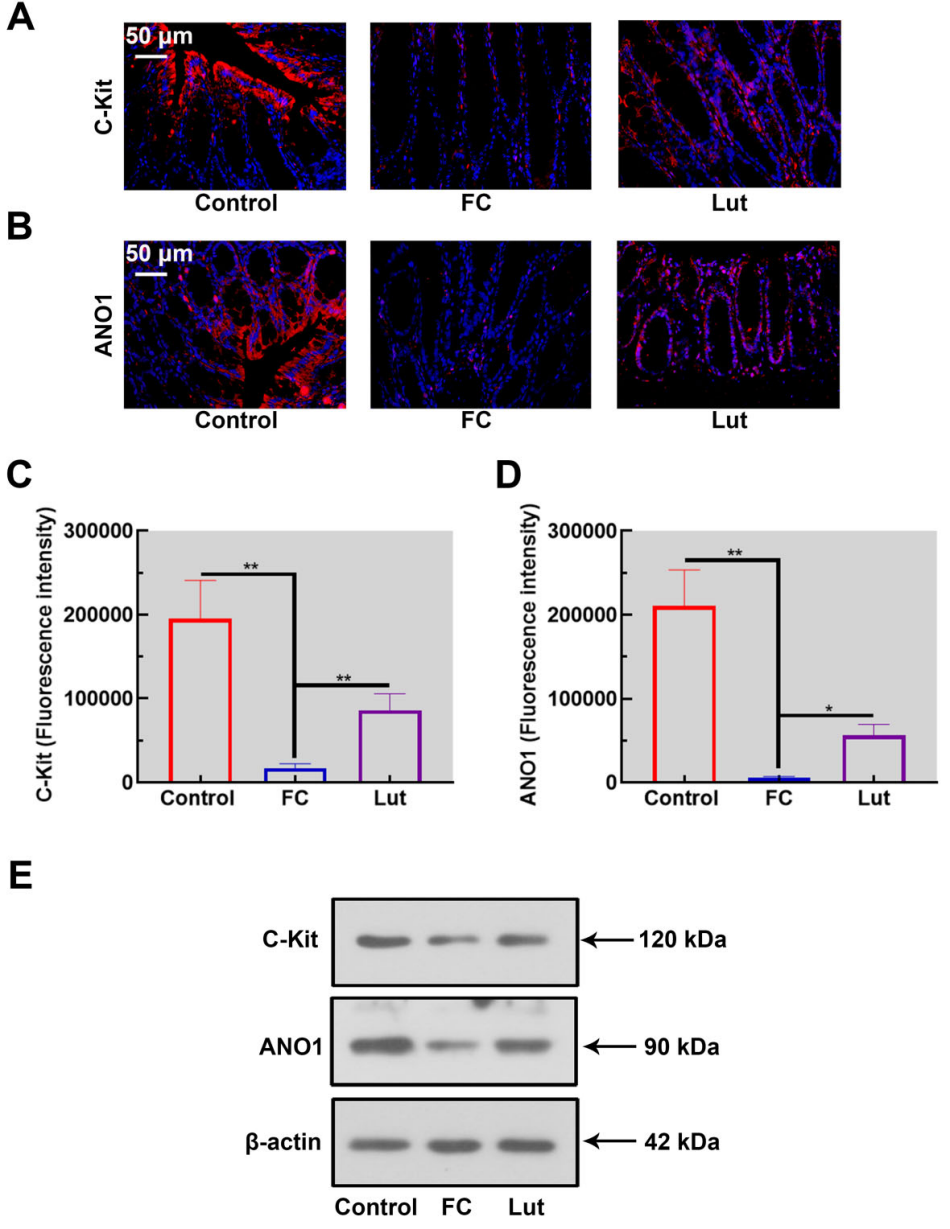
Luteolin (Lut) elevated the expressions of interstitial cells of Cajal (ICC) biomarkers in mice with functional constipation (FC). The expression and distribution of KIT proto-oncogene, receptor tyrosine kinase (C-Kit) (**A** and **C**) and anoctamin-1 (ANO1) (**B** and **D**) in the colon tissues was determined by immunofluorescence (scale bar 50 μm). **E**, Western blotting analysis of C-Kit and ANO1 in the colon of mice. Data are reported as means±SD (n=6). *P<0.05 and **P<0.01 (ANOVA and Tukey's multiple comparisons test).

The expression and distribution of ICC biomarkers were detected by immunofluorescence. Noticeably, both C-Kit and ANO1 were present in longitudinal layers and their levels were significantly decreased in the loperamide-induced FC group. Luteolin effectively increased the protein expressions of C-Kit and ANO1 (Figure 4A-D, P<0.01). Consistent with these results, the immunoblotting analysis indicated loperamide-induced downregulation of C-Kit and ANO1, which were rescued by luteolin treatment (Figure 4E). Therefore, Luteolin increased ICC biomarker levels in the colon of FC mice.

### Luteolin suppressed loperamide-induced upregulation of AQPs

**Figure 5:**
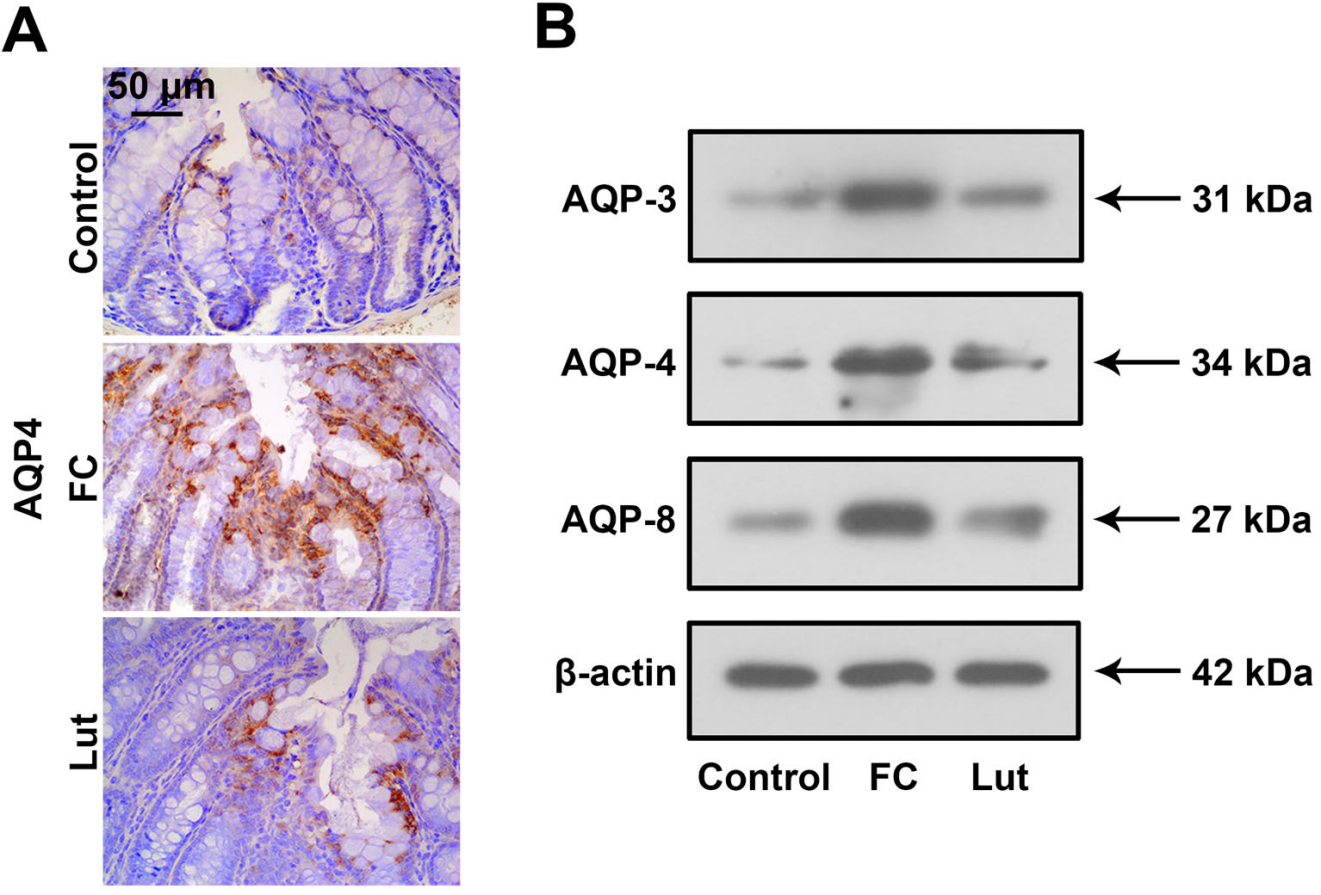
Luteolin (Lut) inhibited the upregulation of aquaporins (AQPs) of mice with functional constipation (FC). **A**, The expression of AQP-4 in the colon tissues was tested by immunohistochemistry (scale bar 50 μm). **B**, Western blotting analysis of AQP-3, AQP-4, and AQP-8 in the colon of mice (n=6).

Protein levels of AQPs, including AQP-3, AQP-4, and AQP-8, in the colon of mice were examined. As shown in Figure 5A, there was weak AQP-4 expression in the colon of the mice from the control group. Nevertheless, large quantities of AQP-4 were observed in the FC group, whereas luteolin decreased the protein expression of AQP-4. In line with the alterations of AQP-4, induction of AQP-3 and AQP-8 were inhibited by luteolin treatment in FC mice (Figure 5B). Hence, luteolin administration decreased levels of AQPs in the colon of FC mice.

## Discussion

**Figure 6:**
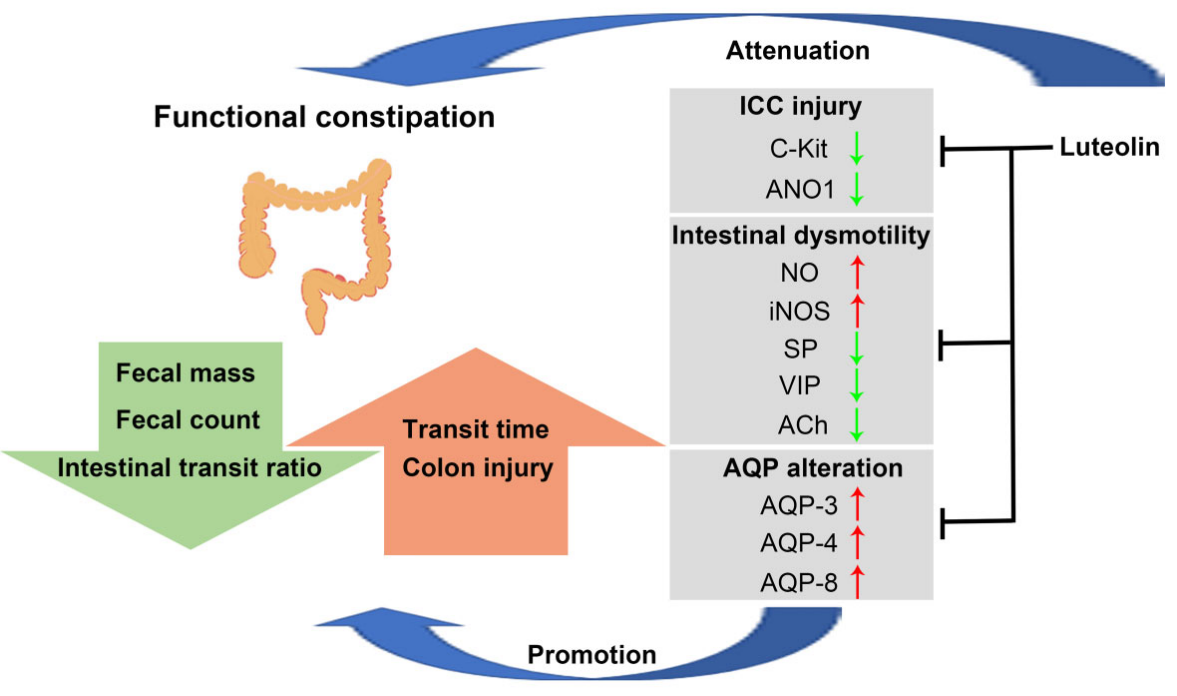
Schematic model of luteolin-alleviated functional constipation (FC). Luteolin balanced the levels of intestinal motility-related biomarkers, promoted intestinal motility, and protected against interstitial cells of Cajal (ICC) damage as well as decreasing aquaporin (AQP) expression, subsequently mitigating FC. NO: nitric oxide; iNOS: inducible nitric oxide synthase; SP: substance P; VIP: vasoactive intestinal polypeptide; ACh: acetylcholine.

In this study, *in vivo* experiments were used to evaluate the effects of luteolin on colon function, ICC biomarkers, and levels of AQPs in the colon of FC mice. Our findings indicated that luteolin reversed the reduction in defecation frequency, fecal water content, and intestinal motility induced by loperamide. We also observed that luteolin could partially restore the effects of loperamide on ICC and the elevated levels of AQPs in the colon of FC mice. The present study firstly showed that luteolin increased intestinal motility, promoted evacuation, and improved ICC injury and inhibited AQP expression, subsequently improving FC (Figure 6).

Previous publications highlighted that FC was associated with abnormal colon function, especially dysmotility of the colon. Notably, normal intestinal motility is mediated by the enteric nervous system, which is regulated by various intestinal motility-related biomarkers. In the intestinal tract, SP acts as an excitatory neurotransmitter, activating intestinal motility (21). VIP, regarded as a mediator in the intestinal peristaltic reflex, participates in inhibitory neuromuscular transmission (22). The reduction of SP and VIP was involved in the enteric nervous system dysfunction in patients with slow transit constipation (23). Additionally, ACh is the primary enteric excitatory neurotransmitter that affects nerves, mucosae, and smooth muscles in the colon (24). It has been reported that a constant decrease of enteric neurons might give rise to a decline of neurotransmitters, including SP and VIP, which results in alterations in the enteric nervous system, subsequently impairing intestinal motility and causing constipation. These effects are mediated by NO production (25). NO was also reported to be the main inhibitory neurotransmitter that decreases intestinal motility in the colon (26). Loperamide-induced enhancement of NO production was reported in a recent study (27). In line with previous studies, the expression of neuronal protein HuC/D and the levels of SP, VIP, and ACh in the colon tissues exhibited a significant decrease after loperamide administration. However, a striking increase of NO and iNOS was found in the colon of FC mice. Luteolin had beneficial effects on restoring the balance of neurotransmitters and maintaining colon function, mitigating FC.

ICC are the most crucial cells regulating intestinal motility, both as intermediates and pacemakers in the human intestinal tract (28). ICC are linked to the electrical pacemaker activity generation that results in spontaneous intestinal contractions (29). C-Kit, a tyrosine kinase receptor, is a biomarker of ICC and is vital for its differentiation and development (29). Moreover, ANO1 is a calcium-activated chloride channel and another marker of ICC (30). Different from C-Kit, ANO1 is not associated with ICC differentiation but is necessary for pacemaker activity of ICC (31). Furthermore, it was reported that the colon tissue of patients with slow transit constipation contained significantly fewer C-Kit- and ANO1-positive ICC than the controls (32). The levels of ICC biomarkers (C-Kit and ANO1) were decreased in the intestinal tissue of mice under loperamide treatment (33). Consistent with these findings, we confirmed that the levels of C-Kit and ANO1 were decreased and indicated a reduction in ICC. Even more importantly, the above alterations were restored by luteolin treatment. It had been previously reported that increased NO in the gastrointestinal tract decreases C-Kit expression and impairs pacemaker function (34). We speculated that the elevation in C-Kit and ANO1 by luteolin administration might prevent aggravation of FC via inhibiting NO production. Elevated C-Kit and ANO1 might maintain ICC function and improve intestinal motility, improving FC.

AQPs are expressed in various tissues in humans and 13 types are currently known: AQP0 to AQP12 (35). Increasing evidence supports that abnormally high expression of the above AQPs could cause a reduction in intestinal juice secretion and excessive colonic water absorption, thus causing constipation (36). Recent studies showed that AQP expression is associated with the activated cyclic adenosine monophosphate (cAMP)-protein kinase A (PKA) signaling pathway, which is connected with intestinal relaxation and downregulation of intestinal motility in FC (37,38). Interestingly, luteolin could inhibit the activities of cAMP and PKA (39,40). It was not surprising therefore to find in this study that luteolin reversed loperamide-induced upregulation of AQPs in the colon and improved FC in mice. The link between luteolin and the cAMP/PAK/AQP signaling system should be investigated in future studies.

In conclusion, the palliative effect of luteolin was associated not only with the elevated expression of gastrointestinal motility-related neurotransmitters but also with the recovery of ICC in the colon of FC mice. Moreover, the effects of luteolin on the transcellular water movement by epithelial colonocytes might involve AQP regulation in FC. Thus, there was powerful evidence that luteolin may be used as a potential novel drug for FC therapy.
